# Patient Care Teams in treatment of diabetes and chronic heart failure in primary care: an observational networks study

**DOI:** 10.1186/1748-5908-6-66

**Published:** 2011-07-03

**Authors:** Jan-Willem Weenink, Jan van Lieshout, Hans Peter Jung, Michel Wensing

**Affiliations:** 1 Scientific Institute for Quality of Healthcare, Radboud University Nijmegen Medical Centre, P.O. Box 9101, 6500 HB, Nijmegen, the Netherlands

## Abstract

**Background:**

Patient care teams have an important role in providing medical care to patients with chronic disease, but insight into how to improve their performance is limited. Two potentially relevant determinants are the presence of a central care provider with a coordinating role and an active role of the patient in the network of care providers. In this study, we aimed to develop and test measures of these factors related to the network of care providers of an individual patient.

**Methods:**

We performed an observational study in patients with type 2 diabetes or chronic heart failure, who were recruited from three primary care practices in The Netherlands. The study focused on medical treatment, advice on physical activity, and disease monitoring. We used patient questionnaires and chart review to measure connections between the patient and care providers, and a written survey among care providers to measure their connections. Data on clinical performance were extracted from the medical records. We used network analysis to compute degree centrality coefficients for the patient and to identify the most central health professional in each network. A range of other network characteristics were computed including network centralization, density, size, diversity of disciplines, and overlap among activity-specific networks. Differences across the two chronic conditions and associations with disease monitoring were explored.

**Results:**

Approximately 50% of the invited patients participated. Participation rates of health professionals were close to 100%. We identified 63 networks of 25 patients: 22 for medical treatment, 16 for physical exercise advice, and 25 for disease monitoring. General practitioners (GPs) were the most central care providers for the three clinical activities in both chronic conditions. The GP's degree centrality coefficient varied substantially, and higher scores seemed to be associated with receiving more comprehensive disease monitoring. The degree centrality coefficient of patients also varied substantially but did not seem to be associated with disease monitoring.

**Conclusions:**

Our method can be used to measure connections between care providers of an individual patient, and to examine the association between specific network parameters and healthcare received. Further research is needed to refine the measurement method and to test the association of specific network parameters with quality and outcomes of healthcare.

## Background

Chronic disease represents a significant challenge for health systems, because it requires major changes in the organization of healthcare and in the tasks of many health professionals [1]. Structured clinical management of chronic disease improves health outcomes and efficiency of the healthcare delivery [2]. Providing chronic care has increasingly become the task of a patient care team, rather than an individual health professional [3], and improved team functioning is expected to be associated with better quality and outcomes of healthcare delivery [4,5]. Previous studies identified numerous factors of team functioning associated with team performance in healthcare, though evidence on performance of primary care teams in treatment of chronic disease remains ambiguous [5-7].

It has been suggested that the presence of a central care provider in a team, who acts as a contact point for both patient and other health professionals and takes responsibility for the delegation of care to others on the team, is crucial in achieving optimal outcomes [8,9]. This could optimize the coordination of healthcare delivery and ensure that all necessary expertise and relevant patient information is present to provide effective clinical management. Patients who receive medical care from a team of health professionals may benefit from a wider range of skills. The inclusion of specific individuals, such as a nurse or pharmacist, may ensure that specific elements are more evidence-based [3]. A few field studies showed that the type and diversity of clinical expertise involved was expected to account for improvements in patient care and organizational effectiveness [10,11]. Finally, sharing knowledge in patient care teams could lead to shared practice routines and better coordination of care.

A key aspect of chronic illness care is that it should take a patient-centered focus, meaning that it is respectful of and responsive to individual patient preferences and needs [12]. Ideally, it is characterized by productive interactions between team and patient that consistently provide the assessments, support for self-management, optimization of therapy, and follow-up associated with good outcomes, and these interactions are more likely to be productive if patients are active, informed participants in their care [8]. Previous studies have focused on patient-perceived involvement [13] and communication of teams to patients in general [14]. Actual involvement of individual patients in processes of healthcare delivery was measured less frequently [15].

Network analysis is a quantitative methodology that offers the opportunity to measure and analyze connections between health professionals in a patient care team [16,17]. Pilot studies have examined the feasibility and relevance of network analysis for studying patient care teams in chronic illness care [18,19]. In these pilots, interactions were measured in a generic way. However, networks of health professionals differ across individual patients, even if they have the same disease and same primary care provider. Furthermore, the patient was not included in the networks in these pilots. In addition, associations between network characteristics and healthcare delivery were not yet examined in chronic illness care. Thus, our aim was to measure information exchange networks related to individual patients with a chronic disease, including relevant health professionals and the patient, and to relate network characteristics to aspects of healthcare received.

Our study focused on three specific aspects of healthcare for patients with type 2 diabetes or chronic heart failure (CHF): medical treatment, physical exercise advice, and monitoring. Previous research has shown gaps between recommended practice and healthcare received in these patients [2,20,21], suggesting a potential for improvement. The structure of the networks of information flows between the patient and care providers, and among care providers, was expected to be particularly related to monitoring routines. Monitoring demands an active role of the team [22]. Furthermore, it requires a clear task distribution, knowledge on latest guidelines, and convincement of its benefits. Despite recommendations in prevailing practice guidelines, these benefits remain a topic for continuing debate [23]. Therefore, we expected that social factors would be associated with monitoring routines.

Three specific objectives were defined. A first objective was to test the feasibility of the sampling and measurement procedures, because some previous network studies did not fully report on response rates [18,24]. A second objective was to examine the variation of network characteristics across individual patients, because this would open the possibility that these characteristics are related to relevant outcomes and across chronic conditions. A final objective was to explore associations between specific network characteristics and comprehensive monitoring in these patients, although the size of our study was too small to draw firm conclusions on these associations.

## Methods

### Study design

An observational study was performed for which we invited 30 patients with type 2 diabetes and 30 patients with CHF from three primary care practices. In each practice, we randomly selected 10 patients with diabetes and 10 patients with CHF in the medical record system. Patients with diabetes were selected using available datasets in the practices, patients with CHF were selected with use of the International Classification of Primary Care(ICPC) code. If a patient was physically or mentally incapable to participate, he or she was replaced by the next patient on the list. The ethical committee of Arnhem-Nijmegen waived approval for this study. Patients, general practitioners (GPs), practice nurses, and practice assistants in the participating practices were asked to complete a structured questionnaire. Written informed consent was obtained for collecting data from the patients' medical record.

### Measures

#### Patient questionnaire

Patients were asked to report on the number of disease-specific contacts they had had in the past 12 months concerning medical treatment, physical exercise advice, and disease monitoring, and what health professionals were involved in these contacts. Medical treatment was defined to the participants as any contact related to disease-specific medication (*e.g.*, dosage, application, adverse effects). Physical exercise advice was defined as any contact related to physical exercise or its importance. Disease monitoring was defined as any contact related to disease-specific blood monitoring. Health professionals, both in general practice as outside the practice, were listed by discipline. Other questions concerned general patient and disease characteristics.

#### Medical records

After patients' written informed consent, we extracted information from medical records concerning individual characteristics and received monitoring. Parameters included bodyweight, body mass index, blood pressure, HbA1C (only for diabetes patients), glucose, serum creatinine, potassium, sodium, and lipid values. Medication for diabetes and cardiovascular conditions was also extracted.

#### Care provider questionnaire

Health professionals in the practices were asked about their role in diabetes and CHF care in general, and about their collaboration with other health professionals in medical treatment, physical exercise advice, and disease monitoring. For these three specific activities, they were asked to report on patient-related contact with other disciplines, both inside as outside their practice. Health professionals were listed by discipline.

### Data analysis

We used UCINET 6 for constructing networks and obtaining network parameters, and SPSS 15 for all other analyses. Response rates for both patients and health professionals were determined. We determined reliability of reported connections with other health professionals by examining the proportion of all possible connections that were mutually reported present or absent (called reciprocity coefficients in non-directed networks).

### Construction of networks and network parameters

For each patient, three activity-specific ego-centred networks were constructed, related to medical treatment, physical exercise advice, and disease monitoring. An activity-specific network was only constructed if the patient reported at least one connection with a professional regarding the specific activity. A two-step procedure was used to construct these networks: first, patient questionnaires and medical records were used to identify connections between the patient and health professionals; then care provider questionnaires were used to identify connections between health professionals, defining a connection if either one or both of the health professionals reported to be connected.

If a patient had contact with a health professional within a general practice (*e.g.*, GP), all health professionals in that practice were included in the constructed network. If a health professional was involved in an activity-specific network (*e.g.*, concerning medical treatment), this professional was included in the other activity-specific networks of this patient as well.

If the response of a health professional was missing, it was substituted by the response of the other individuals in the practice. We filled in a zero indicating no contact, if both individuals did not provide information on their connection. This method is commonly used in network analysis [25], though its appropriateness for this specific context has not been tested. A 'zero' in the data files therefore referred to absence of a connection, or absence of data on presence of a connection.

### Network parameters and hypotheses

We examined a number of specific network parameters, which we hypothesised to be related to healthcare delivery and outcomes.

Size and diversity are the number of involved health professionals and different disciplines. A high number of involved health professionals could hinder coordination of care for an individual patient. Multiple involved disciplines, however, could be beneficial because of the availability of a wider range of skills [5].

Density is the proportion of all possible connections in a network that are actually present. In a dense network, information can flow quickly between most individuals. It may also be associated with a number of cognitive social processes, which result in positive intentions in team members to use the information in daily practice. This could contribute to more evidence-based and more standardized practice patterns [26].

Network centralization is a measure that expresses to what extent a network is organized around a single person. It has been suggested that the presence of a central care provider in chronic illness care is crucial to achieve optimal outcomes [8].

The degree centrality coefficient is the proportion of all possible connections that are actually present for an individual. We computed degree centrality coefficients for the patient and for the most central health professional. The discipline of the most central health professional was also noted. A high centrality of the health professional can contribute to coordination of care through connection with many other involved health professionals. When this central health professional is one with high expertise (in a general practice usually a GP), knowledge on the best possible care can flow through the patient care team. Furthermore, initiatives on improving healthcare more often focus on a central role for the patient in its own care process [8]. We think active involvement of a patient will result in a comprehensive monitoring policy in that patient.

Overlap is the proportion of present and absent ties in an index activity-specific network that are also present in another activity-specific network. Medication, advice, and monitoring overlap numbers of patients were obtained to see if different health professionals were involved in different aspects of the care process. It was expected that a high overlap could contribute to coordination of care, because involved health professionals will have knowledge of the entire care process of a patient, instead of just a smaller part.

### Descriptive and comparative analysis

Descriptive statistics of network parameters and clinical management in the previous 12 months were computed for the two chronic conditions. For follow-up and identifying co-morbidity, it is important to establish body mass index (BMI)/weight, systolic blood pressure, and creatinine values at least once a year in patients with diabetes, as well as with CHF [27,28]. We computed a variable for received comprehensive monitoring that indicated if all three values were obtained at least once in the previous 12 months. Descriptive statistics for both conditions were computed, as well as network parameters for both groups of monitoring received (not all monitored/all monitored). Significance of differences in network parameters between the two conditions, and between two monitoring groups, was tested using the Mann-Whitney test.

## Results

### Feasibility

In one practice, a total of seven CHF patients could be identified. Therefore, a total of 57 patients was invited to participate, of whom 32 patients completed the questionnaire and gave permission for collecting data from their medical record. Patient response rates varied between practices and the two chronic conditions (Table 1). Response rates of health professionals (range: 80 to 100% per practice) and reciprocity coefficients in the three networks of healthcare professionals were high (range: 0.667 to 0.857 per practice).

**Table 1:** Response rates per practice and condition, and reciprocity of health professionals

		Practice 1	Practice 2	Practice 3	Total
**Patients**	Total	45.0% (9/20)	80.0% (16/20)	41.2% (7/17)	**56.1% (32/57)**
	Diabetes	40.0% (4/10)	90.0% (9/10)	50.0% (5/10)	**60.0% (18/30)**
	Chronic heart failure	50.0% (5/10)	70.0% (7/10)	28.6% (2/7)	**51.9% (14/27)**
**Health professionals**		100.0% (6/6)	100.0% (6/6)	80.0% (8/10)	**90.9% (20/22)**
	Reciprocity^a^	0.667	0.800	0.857	

Reciprocity is the proportion of all possible connections that are mutually reported present or absent by health professionals

**Figure 1:**
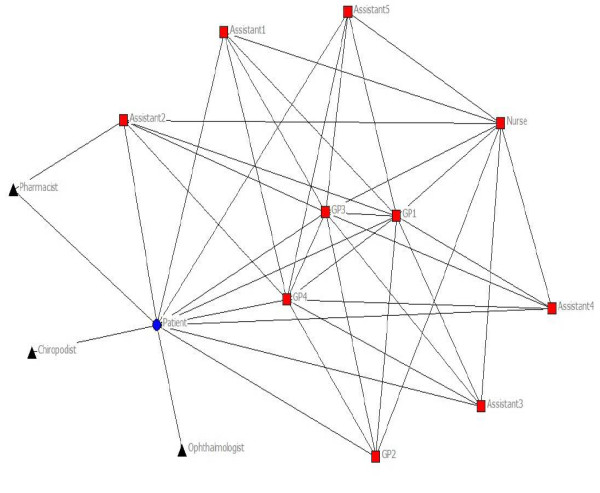
**Network of a patient with diabetes for medical treatment**. Circle: patient; square: health professional in practice; triangle: health professional outside practice. Included for illustration of the method used. The network illustrates the patient and the health professionals involved. Lines resemble a connection between two specific individuals.

**Figure 2:**
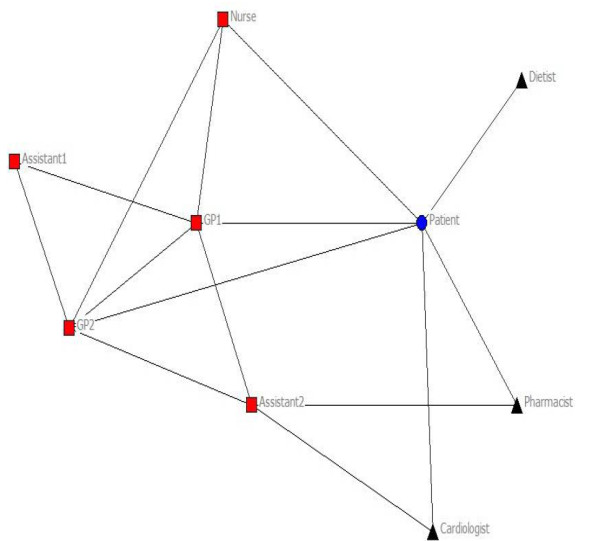
**Network of a patient with CHF for medical treatment**. Circle: patient, square: health professional in practice, triangle: health professional outside practice. Included for illustration of the method used. The network illustrates the patient and the health professionals involved. Lines resemble a connection between two specific individuals.

In three out of 32 patients, no connections with health professionals could be deduced from either questionnaires or medical record, so these patients were excluded from further analysis. Of the theoretical maximum of 87 activity-specific networks, a total of 72 networks were identified: 24 for medical treatment, 20 for physical exercise advice, and 28 for disease monitoring. Four patients with CHF had received all treatment in hospital rather than primary care in the previous 12 months. These patients were excluded for further analysis, leaving a total number of 25 patients with 63 networks: 22 for medical treatment, 16 for physical exercise advice, and 25 for disease monitoring. Table 2 illustrates patient characteristics of our study population. Figure 1 and 2 illustrate networks for medical treatment of a patient with diabetes and a patient with CHF.

**Table 2:** Patient characteristics study population (n = 25)

Disease	Diabetes	72% (N = 18)
	Chronic heart failure	28% (N = 7)

**Gender**	Male	44% (N = 11)

	Female	56% (N = 14)

**Age**	Mean	72.83 (sd = 10.72)

**Ethnicity**	Dutch	100% (N = 25)

**Living situation**	Alone	56% (N = 14)

	Spouse	36% (N = 9)

	Spouse and children	8% (N = 2)

**Education**	None	4% (N = 1)

	Primary	36% (N = 9)

	Secondary	56% (N = 14)

	Higher	4% (N = 1)

### Variation of network characteristics

Table 3 shows the mean and standard deviation of size, diversity, density, centrality, and overlap of activity-specific networks for the total number of patients, as well as differences in mean between patients with diabetes and patients with CHF. Substantial variation existed between individual patients, as well as between diabetes and CHF. Differences were found in size and diversity of networks between diabetes and CHF. For all three activities, more health professionals and disciplines tended to be involved in diabetes, though differences were not found to be significant. Density of networks and the total number of connections tended to be higher for diabetes, though only difference in density of physical exercise advice networks was found to be significant (p = 0.005). The difference in the total number of connections in a network was only found to be significant (p = 0.034) for medical treatment. Network centralization seemed to be equal for medical treatment and monitoring, and showed a (non-significant) difference for physical exercise advice. On all three activities, degree centrality of the most central health professional tended to be higher for diabetes, though this difference was significant for physical exercise advice only. The patients' degree centrality tended to be higher for physical exercise advice only, though no significant difference was found. Overlap values did not vary much between the chronic conditions.

**Table 3:** Mean and standard deviation of network parameters, and differences between chronic conditions

		Total	Standard deviation	Diabetes	Chronic heart failure	Significance of difference between conditions
**Size and diversity**						

Treatment	Number of professionals	8.14	2.336	8.56	7.00	0.133

	Different disciplines	4.77	1.232	5.00	4.17	0.170

Advice	Number of professionals	7.88	2.778	8.54	5.00	0.080

	Different disciplines	4.69	1.401	4.92	3.67	0.257

Monitoring	Number of professionals	7.64	2.059	7.94	6.86	0.336

	Different disciplines	4.40	1.080	4.56	4.00	0.271

**Density**						

Treatment	Density	0.4803	0.1048	0.4900	0.4543	0.376

	Number of connections	19.05	10.96	21.06	13.67	0.034

Advice	Density	0.3520	0.1284	0.3906	0.1845	0.005

	Number of connections	16.62	9.44	18.46	8.67	0.121

Monitoring	Density	0.4659	0.1527	0.4896	0.4049	0.348

	Number of connections	18.52	11.91	20.56	13.29	0.192

**Centrality**						

Treatment	Network centralization	51.85	12.80	52.87	49.14	0.652

	Most centralized health prof.	GP		GP	GP	

	Degree of most central health prof.	85.44	15.44	87.31	80.46	0.337

	Patient's degree centrality	52.72	23.88	53.06	51.83	0.679

Advice	Network centralization	41.03	13.08	42.44	34.90	0.593

	Most centralized health prof.	GP		GP	GP	

	Degree of most central health prof.	63.52	17.57	68.47	42.06	0.027

	Patient's degree centrality	52.26	22.56	55.43	38.49	0.225

Monitoring	Network centralization	50.13	12.05	50.51	49.16	0.847

	Most centralized health prof.	GP		GP	GP	

	Degree of most central health prof.	83.69	14.39	86.12	77.43	0.085

	Patient's degree centrality	52.60	23.81	53.14	51.21	0.801

**Overlap**						

	Treatment - advice	0.7571	0.0956	0.7643	0.7283	0.615

	Treatment - monitoring	0.8747	0.0673	0.8796	0.8617	0.788

	Advice - monitoring	0.7653	0.0731	0.7617	0.7810	1.000

Table 4 shows the clinical management in the previous 12 months for both chronic conditions. The total number of disease-specific contacts was higher for diabetes patients, and so was the number of contacts for blood value monitoring. Variation existed on received monitoring.

**Table 4:** Clinical management in the previous 12 months

	Diabetes	CHF
**Mean number of contacts**		

Disease specific consultation	10,17	3,71

Blood value monitoring	4,44	3,14

**Monitoring in % (N)**		

Weight	94 (17/18)	43 (3/7)

Body Mass Index	83 (15/18)	29 (2/7)

Systolic blood pressure	100 (18/18)	86 (6/7)

HbA1C	83 (15/18)	-

Glucose	78 (14/18)	57 (4/7)

Creatinine	44 (8/18)	86 (6/7)

Potassium	39 (7/18)	71 (5/7)

Sodium	22 (4/18)	71 (5/7)

Total cholesterol	50 (9/18)	57 (4/7)

HDL	50 (9/18)	57 (4/7)

LDL	44 (8/18)	57 (4/7)

Triglycerides	61 (11/18)	57 (4/7)

**Treatment in % (N)**		

No treatment	11 (2/18)	-

Diet	28 (5/18)	-

Oral medication	67 (12/18)	-

Insulin	22 (4/18)	-

Antihypertensive	89 (16/18)	100 (7/7)

Lipid-lowering medication	78 (14/18)	57 (4/7)

ACE-inhibitor	-	57 (4/7)

Beta blocker	-	86 (6/7)

Furosemide + ACE-inhibitor in comb. w/NSAID	-	43 (3/7)

### Association network parameters with received monitoring

Ten out of 25 patients (40%) received monitoring on BMI/weight, systolic blood pressure, and creatinine. Table 5 shows values of network parameters for patients who did receive and did not receive this comprehensive monitoring. Differences were found in size of networks, network centralization of medical treatment and advice, degree centrality of health professionals and patients, and in overlap of medical and advice networks. Centrality of the most central health professional was positively associated with monitoring received, while the association of patient centrality with monitoring received was ambiguous for specific activities. A positive association was observed for physical exercise, while a negative association was found for monitoring and no association was observed for medical treatment. Only differences in size of medical and advice networks, and the number of connections in advice networks, were found to be significant.

**Table 5:** Network characteristics by groups of monitoring (BMI/Weight, systolic blood pressure, and creatinine)

		No comprehensive monitoring	Comprehensive monitoring	Significance
**Size and diversity**				

Treatment	Number of professionals	7,08	9,67	0,025

	Different disciplines	5,08	4,33	0,222

Advice	Number of professionals	6,56	9,57	0,029

	Different disciplines	5,00	4,29	0,299

Monitoring	Number of professionals	6,87	8,80	0,085

	Different disciplines	4,67	4,00	0,160

**Density**				

Treatment	Density	0,4707	0,4941	0,566

	Number of connections	13,31	27,33	0,078

Advice	Density	0,3416	0,3653	0,662

	Number of connections	10,89	24	0,009

Monitoring	Density	0,4600	0,4748	0,817

	Number of connections	13,67	25,80	0,265

**Centrality**				

Treatment	Network centralization	49,72	54,93	0,314

	Most centralized health prof.	GP	GP	

	Degree of most central health prof.	82,54	89,63	0,381

	Patient's degree centrality	53,44	51,69	0,987

Advice	Network centralization	37,58	45,46	0,365

	Most centralized health prof.	GP	GP	

	Degree of most central health prof.	61,57	66,01	0,897

	Patient's degree centrality	48,33	57,30	0,518

Monitoring	Network centralization	50,05	50,25	0,967

	Most centralized health prof.	GP	GP	

	Degree of most central health prof.	82,25	85,84	0,604

	Patient's degree centrality	56,48	46,78	0,672

**Overlap**				

	Treatment - advice	0,8014	0,7066	0,076

	Treatment - monitoring	0,8807	0,8661	0,910

	Advice - monitoring	0,7838	0,7416	0,391

## Discussion

This study showed that it is possible to construct networks of health professionals for individual patients with diabetes and CHF using simple structured questionnaires for patients and health professionals, and patients' medical records. Our study population was small, because we aimed to develop and test the method before applying it on a larger scale. Of all invited patients, about 50% was willing to participate. The reliability of the reported connections (in terms of connections' reciprocity) was high for health professionals. Network characteristics varied substantially across individual patients, as well as across chronic conditions. We observed an association between a high degree centrality of the most central health professional and comprehensive disease monitoring, but further research is needed to draw firm conclusions.

Some limitations of this study have to be mentioned. The study was based on a small convenience sample of patients from a few general practices. Differences in response rate were found between the three primary care practices. Due to the short timeframe, study participants were not sent a reminder; However, this is recommended for future studies to elevate response rates. Furthermore, the Dutch healthcare system includes a well-developed primary care system with financial incentives to provide chronic care in primary care settings, so the results cannot be generalized to other settings. The selection of patients with CHF might not be completely appropriate due to inaccurate use of ICPC coding. Care provider questionnaires focused on patient-related contacts with other professions in general, not specific contacts for each individual patient. Asking for specific contacts would give a more accurate network for each individual patient; however, it would become more time consuming and therefore less feasible. Furthermore, health professionals were grouped by discipline, not by name individually. This could result in overestimation of connections when more than two health professionals of a discipline are involved in a practice. Reporting contact with that discipline will result in a connection with all health professionals of that discipline, where only one of those health professionals might be meant. Finally, connections with disciplines outside the practice (*e.g.*, physiotherapist) were constructed as one health professional, where in reality more health professionals might be involved per discipline. This could result in underestimation of the total number of involved health professionals.

From a clinical perspective, it is worth mentioning that the variation in our outcome 'disease monitoring' was mainly related to varying levels of creatinine testing, *i.e.*, monitoring of kidney functioning. Clinical research has confirmed the relevance of this in both diabetes patients and CHF patients. In diabetes, testing for creatinine is important in identifying affected kidney functioning due to damaged blood vessels and nerves, resulting in higher risk for renal failure and cardiovascular diseases. In CHF, kidney functioning may be limited because of an affected blood circulation, and creatinine testing provides an important measure to observe disease development and effectiveness of medication [27,28]. Periodic monitoring could therefore be beneficial for a patients' health status, and may help to reduce healthcare costs by reducing numbers of hospital admissions [23].

For most patients, a GP was the most central health professional for all three specific activities. Previous research suggested a positive association between a central network position and knowledge transfer [29]. A central position of a health professional with high expertise could therefore be of importance to the team's knowledge and skills, and as a result enhance efficiency of care delivery and clinical outcomes. The degree centrality of the most central health professional varied across chronic conditions and monitoring groups. For the latter, differences were small, but a positive association was observed between higher degree centrality and receiving comprehensive disease monitoring. In addition, network centralization seemed to be positively associated with received monitoring for medical treatment and physical exercise advice. This could indicate a beneficial influence of a central health professional on coordination of practice routines and delegation of care to the team [8]. While this finding is not entirely new, the added value of network analysis was that it provided a quantitative measure of the 'centrality' of the central health professional. The method used in this study did not examine individual roles and performance of professionals comprehensively, but focused on the presence of a central care provider based on centrality degree. Previous research associated 'leadership clarity' with commitment to excellence and clear team objectives [9], which could also enhance efficiency of care delivery. Further research should examine specific individual roles of professionals (*e.g.*, association between central position and leadership) in a network, and their relation with received healthcare.

Previous research has shown that patient perceptions of involvement were associated with higher enablement, particularly of the patient highly preferred to be involved [30]. On the other hand, receiving highly structured chronic care was associated with lowered enablement in another study [31]. In the current study, we used the patients' position in the network of connections among health professionals to determine their role in healthcare delivery. Although a substantial variation was observed with respect to patients' degree centrality, we did not identify clear patterns with respect to associations with disease monitoring. Thus the potentially beneficial influence of a highly central role of the patient was not confirmed. Given the limitations of our study, we recommend further research to explore the impact of patients' position in the network on delivery and outcomes of healthcare. This research should take into account that the patients' role in healthcare delivery encompasses more than contacts with health professionals (*e.g.*, self-management).

A number of other network characteristics were examined in our study. Previous research has associated the diversity of clinical expertise in a team with better-perceived team effectiveness [32], and it is expected to account for improvements in patient care and organizational effectiveness [10,11]. Our results showed small differences in diversity of clinical expertise, though it tended to be slightly higher for patients who did not receive comprehensive monitoring. It must be noted that obtained data on interactions between health professionals concerned practice in general and not specific patients, and therefore most obtained network parameters were not independent. Size of networks, for example, was found to be strongly related to practice size. The positive association of network size with monitoring received might therefore actually reflect the association of practice size with quality of chronic disease management found in earlier research [33]. Other than testing for significance of differences, we did not perform statistical analyses on the data due to the low number of patients. Future research could focus on multi-level analysis of network parameters to test their association with healthcare delivery.

The application of network analysis on healthcare delivery by patient care teams provides a new framework for examining organization of chronic care. Our pilot study combined patient and health professional perspectives to reflect chronic care practice, and is, to our best knowledge, the first to examine the relation between specific network parameters and clinical functioning of a patient care team for individual patients. This method could potentially identify improvements of care for individual patients, as well as improvements for the organization and effectiveness of patient care teams in general, though research is needed on the association between network structure, received healthcare, and actual clinical outcomes, and on possibilities to change networks of patient care teams. Our findings support undertaking further research to refine the measure method and to examine associations between network parameters and received healthcare.

## Competing interests

The authors declare that they have no competing interests. Michel Wensing is an Associate Editor of Implementation Science. All decisions on this manuscript were made by another senior Editor.

## Authors' contributions

JW designed the study, was responsible for data collection and data analysis, and wrote the paper. JVL and HPJ coordinated data-collection, provided feedback, and approved the final manuscript. MW designed the study, supervised data-analysis, and contributed to the paper. All authors have read and approved the final manuscript.

## References

[B1] Wagner EH Austin BT Von Korff M Organizing care for patients with chronic illness Milbank Q 1996 74 511 544 10.2307/3350391 8941260

[B2] Weingarten SR Henning JM Badamgarav E Knight K Hasselblad V Gano A Jr Ofman JJ Interventions used in disease management programmes for patients with chronic illness-which ones work? Meta-analysis of published reports BMJ 2002 325 925 10.1136/bmj.325.7370.925 12399340 PMC130055

[B3] Wagner EH The role of patient care teams in chronic disease management BMJ 2000 320 569 572 10.1136/bmj.320.7234.569 10688568 PMC1117605

[B4] Stevenson K Baker R Farooqi A Sorrie R Khunti K Features of primary health care teams associated with successful quality improvement of diabetes care: a qualitative study Fam Pract 2001 18 21 26 10.1093/fampra/18.1.21 11145623

[B5] Bosch M Faber MJ Cruijsberg J Voerman GE Leatherman S Grol RP Hulscher M Wensing M Review article: Effectiveness of patient care teams and the role of clinical expertise and coordination: a literature review Med Care Res Rev 2009 66 5S 35S 10.1177/1077558709343295 19692553

[B6] Haward R Amir Z Borrill C Dawson J Scully J West M Sainsbury R Breast cancer teams: the impact of constitution, new cancer workload, and methods of operation on their effectiveness Br J Cancer 2003 89 15 22 10.1038/sj.bjc.6601073 12838294 PMC2394209

[B7] Poulton BC West MA The determinants of effectiveness in primary health care teams Journal of Interprofessional Care 1999 13 7 18 10.3109/13561829909025531

[B8] Wagner EH Austin BT Davis C Hindmarsh M Schaefer J Bonomi A Improving chronic illness care: translating evidence into action Health Aff (Millwood) 2001 20 64 78 10.1377/hlthaff.20.6.64 11816692

[B9] West MA Borrill CS Dawson JF Brodbeck F Shapiro DA Haward B Leadership clarity and team innovation in health care The Leadership Quarterly 14 393 410

[B10] Lemieux-Charles L McGuire WL What do we know about health care team effectiveness? A review of the literature Med Care Res Rev 2006 63 263 300 10.1177/1077558706287003 16651394

[B11] Xyrichis A Lowton K What fosters or prevents interprofessional teamworking in primary and community care? A literature review Int J Nurs Stud 2008 45 140 153 10.1016/j.ijnurstu.2007.01.015 17383655

[B12] Gerteis M Edgman-Levitan S Daley J Delbanco T Through the patient's eyes: understanding and promoting patient-centered care 1993 San Fransisco, Calif.: Jossey-Bass Inc

[B13] Stewart M Brown JB Donner A McWhinney IR Oates J Weston WW Jordan J The impact of patient-centered care on outcomes J Fam Pract 2000 49 796 804 11032203

[B14] Audet AM Davis K Schoenbaum SC Adoption of patient-centered care practices by physicians: results from a national survey Arch Intern Med 2006 166 754 759 10.1001/archinte.166.7.754 16606812

[B15] Elwyn G Edwards A Mowle S Wensing M Wilkinson C Kinnersley P Grol R Measuring the involvement of patients in shared decision-making: a systematic review of instruments Patient Educ Couns 2001 43 5 22 10.1016/S0738-3991(00)00149-X 11311834

[B16] Cott C 'We decide, you carry it out': a social network analysis of multidisciplinary long-term care teams Soc Sci Med 1997 45 1411 1421 10.1016/S0277-9536(97)00066-X 9351158

[B17] Milward HB Provan KG Measuring Network Structure Public Administration 1998 76 387 407 10.1111/1467-9299.00106

[B18] Scott J Tallia A Crosson JC Orzano AJ Stroebel C DiCicco-Bloom B O'Malley D Shaw E Crabtree B Social network analysis as an analytic tool for interaction patterns in primary care practices Ann Fam Med 2005 3 443 448 10.1370/afm.344 16189061 PMC1466914

[B19] Wensing M van Lieshout J Koetsenruiter J Reeves D Information exchange networks for chronic illness care in primary care practices: an observational study Implement Sci 2010 5 3 10.1186/1748-5908-5-3 20205758 PMC2822738

[B20] Seddon ME Marshall MN Campbell SM Roland MO Systematic review of studies of quality of clinical care in general practice in the UK, Australia and New Zealand Qual Health Care 2001 10 152 158 10.1136/qhc.0100152 11533422 PMC1743427

[B21] Bosch M Dijkstra R Wensing M van der Weijden T Grol R Organizational culture, team climate and diabetes care in small office-based practices BMC Health Serv Res 2008 8 180 10.1186/1472-6963-8-180 18717999 PMC2529292

[B22] Von Korff M Gruman J Schaefer J Curry SJ Wagner EH Collaborative management of chronic illness Ann Intern Med 1997 127 1097 1102 9412313 10.7326/0003-4819-127-12-199712150-00008

[B23] Glasziou P How much monitoring? Br J Gen Pract 2007 57 350 351 17504583 PMC2047007

[B24] Keating NL Ayanian JZ Cleary PD Marsden PV Factors affecting influential discussions among physicians: a social network analysis of a primary care practice J Gen Intern Med 2007 22 794 798 10.1007/s11606-007-0190-8 17404798 PMC2219865

[B25] Kossinets G Effects of missing data in social networks Social Networks 2006 28 247 268 10.1016/j.socnet.2005.07.002

[B26] Firth-Cozens J Celebrating teamwork Qual Health Care 1998 7 Suppl S3 7 10339032

[B27] Rutten GEHM Grauw WJC Nijpels G Goudswaard AN Uitewaal PJM Does FEE Heine RJ Ballegooie E Verduijn MM Bouma M Wiersma T, Boukes FS, Geijer RMM, Goudswaard AN NHG-Standaard Diabetes mellitus type 2 NHG-Standaarden voor de huisarts 2009 Bohn Stafleu van Loghum 160 191

[B28] Rutten FH Walma EP Kruizinga GI Bakx HCA Lieshout J Wiersma T, Boukes FS, Geijer RMM, Goudswaard AN NHG-Standaard Hartfalen NHG-Standaarden voor de huisarts 2009 Bohn Stafleu van Loghum 193 212

[B29] Van Wijk R Jansen J Lyles M Inter- and Intra-Organizational Knowledge Transfer: A Meta-Analytic Review and Assessment of its Antecedents and Consequences Journal of Management Studies 2008 45 830 853 10.1111/j.1467-6486.2008.00771.x

[B30] Wensing M Wetzels R Hermsen J Baker R Do elderly patients feel more enabled if they had been actively involved in primary care consultations? Patient Educ Couns 2007 68 265 269 10.1016/j.pec.2007.06.012 17686602

[B31] Wensing M van Lieshout J Jung HP Hermsen J Rosemann T The Patients Assessment Chronic Illness Care (PACIC) questionnaire in The Netherlands: a validation study in rural general practice BMC Health Serv Res 2008 8 182 10.1186/1472-6963-8-182 18761749 PMC2538520

[B32] Shortell SM Marsteller JA Lin M Pearson ML Wu SY Mendel P Cretin S Rosen M The role of perceived team effectiveness in improving chronic illness care Med Care 2004 42 1040 1048 10.1097/00005650-200411000-00002 15586830

[B33] Campbell SM Hann M Hacker J Burns C Oliver D Thapar A Mead N Safran DG Roland MO Identifying predictors of high quality care in English general practice: observational study BMJ 2001 323 784 787 10.1136/bmj.323.7316.784 11588082 PMC57358

